# Acute Limb Ischemia: An Update on Diagnosis and Management

**DOI:** 10.3390/jcm8081215

**Published:** 2019-08-14

**Authors:** Dan-Mircea Olinic, Agata Stanek, Dan-Alexandru Tătaru, Călin Homorodean, Maria Olinic

**Affiliations:** 1 Medical Clinic No. 1, “Iuliu Hatieganu” University of Medicine and Pharmacy, 400006 Cluj-Napoca, Romania; 2 Interventional Cardiology Department, Emergency Clinical Hospital, 400006 Cluj-Napoca, Romania; 3 Department of Internal Diseases, Angiology and Physical Medicine, School of Medicine wih Division of Dentistry in Zabrze, Medical University of Silesia, 41-902 Bytom, Poland

**Keywords:** acute limb ischemia, diagnosis, duplex ultrasound, surgical revascularization, endovascular, thrombolysis, thromboaspiration, mechanical thrombectomy

## Abstract

This review presents an update on the diagnosis and management of acute limb ischemia (ALI), a severe condition associated with high mortality and amputation rates. A comprehensive spectrum of ALI etiology is presented, with highlights on embolism and in situ thrombosis. The steps for emergency diagnosis are described, emphasizing the role of clinical data and imaging, mainly duplex ultrasound, CT angiography and digital substraction angiography. The different therapeutic techniques are presented, ranging from pharmacological (thrombolysis) to interventional (thromboaspiration, mechanical thrombectomy, and stent implantation) techniques to established surgical revascularization (Fogarty thrombembolectomy, by-pass, endarterectomy, patch angioplasty or combinations) and minor or major amputation of necessity. Postprocedural management, reperfusion injury, compartment syndrome and long-term treatment are also updated.

## 1. Introduction

Peripheral artery disease (PAD) is responsible for 12 to 15% of deaths in Europe [1] and is a major burden for the health system. The spectrum ranges from asymptomatic or intermittent claudication to necrosis and limb loss. 

A sudden decrease in limb perfusion that threatens limb viability defines acute limb ischemia (ALI) and represents a major vascular emergency. The clinical presentation is considered to be acute if it occurs within 14 days after symptom onset [2,3]. In contrast to critical limb ischemia (CLI), also called chronic limb-threatening ischemia (CLTI) [4], in which collateral blood supply is often present, ALI threatens limb viability in a very short interval, because there is insufficient time for new blood vessel growth to compensate for the loss of perfusion. The sudden ischemia affects all the metabolically active tissues of the limb: skin, muscles, and nerves. Thus, urgent recognition with prompt revascularization is required to preserve limb viability [2,3].

The incidence of ALI is approximately 1.5 cases out of 10,000 people per year [5]. Complications among ALI patients are high and despite early revascularization, 30-day mortality and amputation rates are between 10 and 15% [3,6]. Also, ALI patients experience increased in-hospital major adverse events including myocardial infarction, congestive heart failure exacerbation, deterioration in renal function and respiratory complications [7].

## 2. Etiology

There are different causes which can lead to ALI, like arterial embolism (30%), arterial thrombosis due to plaque progression and complication (40%), thrombosis of a popliteal aneurysm (5%), trauma (5%) or graft thrombosis (20%) [8]. In another study [9], the incidence of etiologies for ALI is 46% for arterial embolism, 24% for in situ thrombosis, 20% for complex factors and 10% for stent- or graft-related thrombosis. Potential embolic causes related to an acute decrease in limb perfusion are the following: cardiac embolization, aortic embolization (eventually from a thrombosed aneurysm), thrombosed graft, ergotism, hypercoagulable states, paradoxical venous-to-arterial embolism and iatrogenic complications related to endovascular procedures [2].

Native artery occlusions usually occur in the settings of severe, complicated atherosclerotic plaques. In patients with hypercoagulable conditions, in situ thrombosis may develop in a previously normal artery [10].

Some particular conditions are important to be correctly identified, because they do not follow the usual management: embolism from an intracardiac mass (myxoma, vegetation), atheroembolism and calcified debris after transcatheter aortic valve implantation (TAVI), dissections of pelvic and lower extremity arteries.

## 3. Diagnosis

### 3.1. Symptoms and Signs

Symptoms develop in several minutes, to hours or days, and range from new or worsening intermittent claudication to severe rest pain, paresthesia, muscle weakness, paralysis and even gangrene.

The classical description of patients with ALI is grouped into a mnemonic known as the “6 Ps”: pain, pallor, paralysis, pulse deficit, paresthesia and poikilothermia [11].

History taking and physical examination are very important in ALI management. It is often difficult to distinguish an embolic etiology from in situ thrombosis, but it is important because of the different acute phase and long-term treatment options.

In native arterial thrombosis, patients may have prior intermittent claudication or a history of limb revascularization. Also, they have significant comorbidities such as coronary artery disease, history of stroke, diabetes and chronic renal failure. Such patients are aged, frail and vulnerable to bleeding [12]. Nonetheless, the presence of atherosclerotic risk factors (smoking, diabetes, hypertension, high cholesterol, family history) may suggest in situ thrombosis.

Thrombotic complication of an atherosclerotic plaque will make the onset unclear and complaints ambivalent. The gradual progression of atherosclerosis is frequently accompanied by the growth of collateral vessels to distal regions. Acute occlusion in a preconditioned limb may not produce overt ischemia. However, the propagation of the thrombus may induce extensive ischemia.

Embolic occlusions should be suspected in patients with the following features: sudden and severe onset (due to the absence of collateral vessels, the patient is often able to accurately time the moment of the event), prior history of embolism, arrhythmia suggesting atrial fibrillation, known embolic source (cardiac, aneurysm) and no history of intermittent claudication [2].

Acute arterial occlusion leads to intense vascular spasm and the limb will appear “marble” white. Over the next hours, the vessels relax, and the skin fills with deoxygenated blood, leading to a mottled aspect that blanches on pressure [13].

By careful and systematic vascular examination, many causes of ALI can be discovered in a timely manner. The bilateral palpation of the groin, knee and ankle pulses can reveal the site of the occlusion and rhythm disorders, like atrial fibrillation. A unilateral pulse deficit with normal contralateral pulse examination suggests embolism. A bilateral pulse deficit suggests atherosclerotic complication. The vascular examination should include the palpation of the brachial, radial and ulnar arteries in search for possible access sites and multi-site embolism. If there is a doubtful pulse status, the Doppler probe should be used to look for arterial signals. Sensorial capabilities and motor deficit should be assessed at first medical contact and re-evaluated on a regular basis.

The severity of ALI is graded according to the Rutherford classification (Table 1) [14] that plays a major role in decision making. A viable limb mandates urgent imaging as well as the assessment of significant co-morbidities.

The Rutherford classification highlights the prognosis of the affected limb, based on physical examination: skin color, venous filling, motor and sensory function. Also, it includes the presence of Doppler flow signals in pedal arteries and popliteal veins.

### 3.2. Differential Diagnosis

ALI should be distinguished from CLI, in which the duration of symptoms exceeds 2 weeks and is usually much longer. Other conditions include connective-tissue diseases, thromboangiitis obliterans, and vasculitides. Diseases which can mimic ALI or can cause secondary ischemia are aortic dissection involving the iliac vessels, phlegmasia coerulea dolens (deep-vein thrombosis with severe leg swelling compromising arterial flow), compartment syndrome, trauma, systemic shock and the use of vasopressor drugs. Non-ischemic causes of limb pain are acute gout, neuropathy, spontaneous venous hemorrhage and traumatic soft-tissue injury.

### 3.3. Imaging

#### 3.3.1. Duplex Ultrasound

Duplex ultrasound (DUS) is the first imaging choice to assess ALI. It is widely available, has a low cost, is non-invasive, non-irradiant and it takes a relative short time to perform. DUS is useful to assess the anatomic location and the degree of obstruction (complete vs. incomplete). Also, DUS provides important information on hemodynamics (proximal and distal to the obstruction) and is highly useful for the follow up of revascularization procedures [15]. However, in emergency situations without DUS availability, alternative imaging techniques are required.

Evaluation may start with a low-cost continuous wave Doppler pencil probe. Iliac, femoral and popliteal flows are investigated with a 4-MHz probe, while an 8-MHz probe is used for distal arteries. A continuous Doppler provides information on arterial flow presence and character, thus allowing insight into the level of arterial occlusion. A triphasic flow is recognized as normal arterial flow. A pre-occlusive flow has attenuated systolic peak and absent diastolic flow. Distal to an arterial occlusion, flow is typically absent. A continuous systolic/diastolic flow usually suggests an older occlusion, already compensated through collaterals. A continuous Doppler also provides the means for distal pressure measurements.

DUS imaging uses 2D ultrasound (7–10-MHz probes for limbs and 3–5-MHz for abdominal vessels), a color Doppler (largely available recently) and a pulsed wave Doppler. DUS imaging, while operator dependent, provides excellent data at femoral and popliteal levels, while aortic and iliac arteries may be difficult to evaluate in obese patients or due to gas interposition [13].

At the site of an arterial occlusion, DUS shows a non-pulsatile artery, without color flow, with a thrombus within the lumen. DUS can differentiate between a thrombosis on a preexisting chronic and severe stenosis (arterial walls with a significant atherosclerotic plaque) and an embolic event (well delineated, round-shaped thrombus, in the lumen of an artery without significant atherosclerotic burden).

A complete DUS examination should be performed, including the evaluation of arteries proximal and distal to the occlusion, as well as of contralateral arteries. Venous DUS may also be useful for the differential diagnosis and appropriate staging of ALI. Structured imaging databases may be implemented, including the various imaging techniques (cardiac and vascular ultrasound, CT, and digital substraction angiography), in order to provide comprehensive diagnosis tools and facilities for rapid retrieval [16].

#### 3.3.2. Computed Tomography Angiography (CTA) and Magnetic Resonance Angiography (MRA)

CTA and MRA are high-resolution imaging tools, but much of the experience was gathered in patients with CLI or intermittent claudication.

In a meta-analysis, multi-detector computed tomography (MDCT) angiography had a sensitivity and specificity of 96 and 98%, respectively in detecting significant (>50%) aortoiliac stenoses [17]. A similar sensitivity and specificity were reported for the femoropopliteal and below-the-knee arteries. The biggest advantage of CTA is the visualization of calcifications, stents and bypasses. Iodinated contrast agents can worsen renal failure and are generally not indicated in patients with a glomerular filtration rate lower than 60 mL/min.

Gadolinium-enhanced MRA has excellent sensitivity (93–100%) and specificity (93–100%) in comparison to digital substraction angiography (DSA) [18,19]. MRA is useful in patients with allergies or moderate renal failure. Major limitations are the presence of pacemakers or metal implants. Gadolinium is contraindicated in patients with severe renal failure, with a glomerular filtration rate below 30 mL/min [4]. Also, MRA cannot detect arterial calcifications, thus giving limited information for the selection of an anastomotic site.

Patients with ALI may, however, have a limited ability to attend long imaging sessions associated with non-invasive angiography. CTA and MRA are reserved for patients with a non-immediately threatened limb. The use of CTA and MRA for ALI remain very limited.

#### 3.3.3. Invasive Angiogram

DSA was considered for many years the “gold standard” for diagnosis. Because it is an invasive procedure, with a potential risk of complications, DSA should not be used as a first diagnostic tool and should not replace DUS for positive diagnosis of ALI [2,20].

DSA is complementary to DUS and plays an essential role in the therapeutic strategy. Many ALI patients undergo emergency catheter-based interventions following DSA. Surgery or hybrid techniques also rely on DSA. Invasive angiography shows the site of occlusion and the distal arterial tree. It is also useful to distinguish an embolic occlusion from in situ thrombosis. Intravascular imaging, like intravascular ultrasound or optical coherence tomography, are used only in experimental settings [21,22].

## 4. Management

### 4.1. Evaluation of Limb Viability

Patients with clinical suspicion of ALI should be addressed to emergency centers which have a 24-h/365-day vascular team available for diagnosis and management. Because of the high amputation and mortality rates in ALI, there is a need for a 24-h availability of vascular surgery, vascular medicine and/or interventional therapy.

Urgent anticoagulation with unfractionated heparin (UFH) prevents thrombus propagation and preserves microcirculation [23]. Analgesic treatment is often necessary. Routine blood and coagulation tests should be performed. In patients with critically threatened limbs, local venous acidosis should be assessed to predict adverse outcomes and reperfusion injury. If present, acid-base and electrolyte imbalances should be corrected as soon as possible. A careful observation of kidney function before and after revascularization is recommended, especially in older patients or in patients with prior kidney disease.

Auscultation of the heart, chest X-ray and electrocardiography are mandatory in every patient. Cardiac embolism is of particular concern in patients with atrial fibrillation, myocardial infarction, impaired left ventricular function or mechanical heart valves. Echocardiography is useful to evaluate cardiac function, as well as the existence of an embolic source (thrombus, myxoma, vegetation).

In patients with viable (stage I) or marginally threatened (stage IIa) limbs, it is indicated to perform non-invasive imaging (DUS or CTA) to determine the nature and extent of the obstruction and to plan intervention, given the mild-to-moderate risk. In centers where DUS is rapidly available, it should be performed in patients with an immediately threatened limb (stage IIb) in order to guide management and aid prompt revascularization. The availability and duration of imaging techniques must be balanced against the urgency for revascularization.

### 4.2. Treatment

The therapeutic strategy will depend on type of occlusion (thrombus or embolus), location, type of conduit (artery of graft), Rutherford class, duration of ischemia, co-morbidities and therapy-related risks and outcomes.

Different revascularization strategies can be applied, either endovascular or surgical (thrombectomy, bypass and arterial repair). An overview of existing therapeutic options and their scientific result is presented.

#### 4.2.1. Endovascular Techniques

The goal of the catheter-based approach is to restore blood, as quickly as possible, to the threatened limb, with the use of drugs, mechanical devices, or both, using several options for reperfusion: endovascular procedures such as percutaneous catheter-directed thrombolysis (CDT), percutaneous thromboaspiration (PAT), with or without thrombolytic therapy, or percutaneous mechanical thrombectomy (PMT).

##### Catheter-Directed Thrombolysis

In the mid-1990s, three major trials (Rochester study [24], STILE [25], TOPAS [26]) analyzed over 1000 patients with ALI, randomly assigned to CDT or surgical revascularization. Clinical outcomes were similar in the two groups and amputation-free survival rates at 6 months and one year were not significantly different. Patients with symptoms of longer duration (>14 days) had better outcomes after surgery. In the thrombolytic treated group, patients with graft thrombosis benefited more than patients with native artery occlusion. Patients assigned to CDT had lower rates of procedure-related morbidity and mortality compared to the surgery group, at a cost of higher bleeding complications.

Using CDT, complete or partial thrombus resolution, with a satisfactory clinical result, occurs in 75–92% of ALI patients with occluded native vessel, stent or graft [26].

Because of the long time to reperfusion, CDT it is generally not indicated in Rutherford stage IIb. Patients with suspected graft infection, symptom duration >14 days, contraindications to thrombolysis (Table 2) and failure to position the catheter across the thrombus should not undergo CDT. Up to 20% of patients can have a contraindication to thrombolytic therapy [25].

The main thrombolytic agents that are currently in use for ALI are presented in Table 3. However, use might be limited by availability. Systemic thrombolysis has no role in ALI.

During infusion through a multi-hole catheter, patients should be admitted to an intensive care unit, blood count and coagulation profile are mandatory to be periodically measured. Clinical and angiographic examinations should be performed to determine progress. It is very important to conserve limb temperature (e.g., Rooke boot) for optimal thrombolytic action [27]. Distal thrombus embolization commonly occurs as the thrombus is lysed, but the embolized thrombus typically clears during thrombolytic infusion [28].

After the successful restoration of blood flow, angiography is performed to detect pre-existing arterial lesions, which can be managed by endovascular (e.g., stenting) or surgical techniques (e.g., bypass).

Another new technique is ultrasound-accelerated thrombolysis (USAT) which uses sound waves to accelerate thrombolysis. Low-frequency sounds mechanically fragment clots and augment enzymatic fibrinolysis [30].

A recent, multicenter, randomized trial compared standard CDT vs. USAT (EKOS Corporation, Bothell, WA, USA) in ALI treatment. The results showed that patients treated with USAT achieved revascularization 12 h sooner than CDT, with no increase in major adverse effects. Also, USAT required significantly fewer units of thrombolytic agent [31].

Bleeding, related to CDT, occurs most commonly at the catheter-insertion site. Clinically significant hemorrhage occurs in 6 to 9% of patients and intracranial hemorrhage in less than 3% [32]. Factors associated with an increased risk of bleeding include the intensity and duration of CDT, the presence of uncontrolled hypertension, age over 80 years and a low platelet count [33].

Although thrombolysis is effective, 25% of patients require an open procedure, suggesting that patient selection for thrombolysis first strategy instead of open surgery continues to be a clinical challenge [34].

##### Percutaneous Thromboaspiration

It is a low-cost, rapid technique which uses large lumen catheters (6–8F) connected to a syringe. It is used in combination with thrombolysis to reduce procedural time in advanced ischemia. Thromboaspiration alone has been reported to have modest procedural success rates of approximately 30%, but combined with thrombolysis, the primary success rate reached 90%, with a limb salvage rate of 86% [35]. Thromboaspiration is highly effective in treating acute iatrogenic distal embolization during endovascular procedures.

The first-line use of PAT for endovascular treatment of ALI can reduce the need for CDT, with no significant cost difference [36].

Low-profile, dual-lumen, rapid-exchange aspiration catheters are commercially available, such as the Pronto extraction catheter (Teleflex, Morrisville, NC, USA), the Export catheter (Medtronic, Minneapolis, MI, USA), the Xpress-Way extraction catheter (Atrium Medical, Osaka, Japan) and the ASAP catheter (Merit Medical, South Jordan, UT, USA) [37].

##### Percutaneous Mechanical Thrombectomy

The percutaneous removal of a thrombus is often used as first-line therapy for ALI patients. PMT is defined as endovascular thrombus maceration and removal with the use of dedicated percutaneous thrombectomy devices (PTDs). It is mainly indicated in Rutherford stage IIb because the time to reperfusion is significantly shorter than with CDT. Patients with contraindications to thrombolysis and high surgical risk can also benefit from PMT.

In patients at high risk of bleeding, PMT can be used to debulk the thrombus mass before local lysis to shorten the treatment period, thereby limiting the dose of thrombolytic agent needed [37]. PMT may also be used as an adjunctive procedure for incomplete thrombolysis or to treat distal embolic complications following CDT. However, well organized thrombi are still problematic for most PTDs. Distal micro-embolization with subsequent limb loss is the main concern when using PTDs. For this reason, devices with additional fragment aspiration are preferred: Rotarex (Straub Medical AG, Wangs, Switzerland), AngioJet (Boston Scientific, Marlborough, MA, USA) and Trellis (Bacchus Vascular, Santa Clara, CA, USA) [38,39,40]. Vessel perforation and dissection may appear with PMT. The risk of perforation is especially high in calcified arteries.

A number of studies mention very high technical success rates (over 90%) for primary re-opening of infra-aortic vessels with PMT [41,42,43]. Usually, there is a need to perform balloon angioplasty or stent implantation after successful removal of the thrombotic material. The amputation-free survival at 12 months was better after PMT compared to CDT or surgical thrombectomy [23].

A direct prospective comparison between PMT and CDT (a non-randomized, retrospective, single-center study [44]) showed that in patients with ALI, mechanical thrombectomy performed with Rotarex represents a safe and effective alternative to thrombolysis and is associated with a reduced rate of major bleedings, shorter hospitalization durations and lower costs. Another study [45] showed that mechanical debulking using the Rotarex system may be a safe and effective treatment option in case of thrombotic or embolic occlusion of the proximal and mid-portion of crural arteries.

Aspiration thrombectomy with the Indigo system (Penumbra, Alameda, CA, USA) has two key advantages: it does not require the use of lytic agents and it provides immediate flow reestablishment. It can be used when thrombolysis has failed or is contraindicated. The Indigo system promotes active thrombectomy using a powerful vacuum pump that generates substantial suction, enabling aspiration of clots of varying sizes and lengths [46].

Unlike conventional vacuum-based automatic aspiration devices, the Clearlumen-II system (Walk Vascular, Irvine, CA, USA) simultaneously aspirates the thrombus and performs pulse spray thrombolysis with a high-pressure jet of saline [47].

#### 4.2.2. Open Surgery

Patients with an immediately threatened or nonviable limb, bypass graft with suspected infection, or contraindication to thrombolysis should undergo open revascularization. Also, the surgical approach is preferred in patients with ischemic symptoms for over 2 weeks [48].

Surgical procedures in ALI include thrombectomy with a balloon catheter (Fogarty), bypass surgery and adjuncts such as endarterectomy, patch angioplasty, and intra-operative thrombolysis. Frequently, a combination of these techniques is required.

Patients with suspected embolism and an absent femoral pulse ipsilateral to the ischemic limb are best treated by exposure of the common femoral artery bifurcation and balloon-catheter thrombectomy. A recent refinement for thrombectomy is the use of over-the-wire catheters, allowing for selective guidance into distal vessels.

A meta-analysis of six clinical trials (five randomized prospective, one retrospective, including a total of 1773 patients) showed that, in patients presenting with ALI, endovascular and surgical approaches have similar rates of short-term and 12-month mortality, limb amputation and recurrent ischemia [49].

However, open surgery is recommended as the best option for thromboembolism and for patients with Rutherford class IIb. In contrast, endovascular treatment should be the preferred choice of treatment for patients presenting with Rutherford class I and IIa [50].

ALI caused by popliteal aneurysm thrombosis warrants special mention, because major amputation occurs with high frequency in such patients [51]. Diffuse thromboembolic occlusion of all major runoff arteries below the knee is frequently seen, and intra-arterial thrombolysis or thrombectomy may be required to restore flow in a runoff artery before aneurysm exclusion and surgical bypass are performed.

#### 4.2.3. Reperfusion Injury

Reperfusion injury may result after target vessel re-opening, especially in advanced stages of ischemia. Profound limb swelling, with a dramatic increase in compartmental pressures, is the trademark of this phenomenon [52]. Patients experience severe pain and hyperesthesia of the affected limb. The anterior compartment of the leg is the most susceptible to this phenomenon, leading to peroneal nerve dysfunction. The diagnosis is based on clinical findings but can be confirmed if the compartment pressure is over 30 mmHg [52]. The presence or development of compartment syndrome is an absolute contraindication to thrombolysis, so other revascularization techniques should be applied. If the compartment syndrome occurs, surgical fasciotomy is indicated to prevent irreversible neurologic and soft-tissue damage.

#### 4.2.4. Postprocedural and Follow-Up Care

The restoration of a palpable pulse, audible arterial Doppler signals and visible improvement of perfusion suggest treatment success. Dorsiflexion of the foot and sensory function should be assessed after the revascularization procedure to screen for compartment syndrome.

In some cases, perfusion may be incomplete and close postoperative observation is required to monitor the limb status and prepare for amputation. Vasodilators (e.g., nitroglycerin and papaverine) may be useful if there is evidence of vasospasm. In the case of atherosclerotic in situ thrombosis, after clot removal, correction of the underlying arterial abnormality is critical for long-term patency.

Patients with thromboembolism or thrombophilia will need long-term anticoagulation with vitamin K antagonists to prevent future events. Novel oral anticoagulants (dabigatran, apixaban, rivaroxaban, edoxaban) should be considered in patients with non-valvular atrial fibrillation and a cardioembolic etiology for ALI.

Patients with thrombosis complicating atherosclerotic lesions will benefit from long-term treatment with statin and anti-platelet therapy to improve long-term vessel patency and survival [53]. Dual anti-platelet therapy with aspirin and clopidogrel is indicated after stent implantation, for at least one month [54,55].

## 5. Limitations of Available Data and Need for Future Studies

Currently available data have limitations regarding the number of randomized studies using the different management techniques. While surgery is widely used and provides major clinical benefit, the new endovascular techniques are promising but still limited to use in highly specialized centers. Recommendations for ALI registries have been recently published to improve the situation [56].

## Figures and Tables

**Table 1:** Stages of acute limb ischemia (ALI) according to the Rutherford classification [14].

Stage	Prognosis	Findings		Doppler Signal	
		Sensory Loss	Muscle Weakness	Arterial	Venous
I	Limb viable, not immediately threatened	None	None	Audible	Audible
IIa	Limb marginally threatened, salvageable if promptly treated	Minimal (toes)	None	Often inaudible	Audible
IIb	Limb immediately threatened, salvageable with immediate revascularization	More than toes, pain at rest	Mild or moderate	Inaudible	Audible
III	Limb irreversibly damaged, major tissue loss or permanent nerve damage inevitable	Profound, anesthetic	Paralysis (rigor)	Inaudible	Inaudible

**Table 2:** Absolute and relative contraindications to catheter-directed thrombolysis (CDT) (modified after [29]).

**Absolute Contraindications to CDT**
Active bleeding
Intracranial hemorrhage
Presence or development of compartment syndrome
Severe limb ischemia, requiring immediate operative intervention
For streptokinase: prior administration of streptokinase
**Relative Contraindications to CDT**
Uncontrolled hypertension > 180/110 mmHg
Puncture of non-compressible vessel
Intracranial tumor
Ischemic cerebrovascular event < 2 months
Neurosurgery or head trauma within past 3 months
Gastrointestinal bleeding < 10 days
Hepatic failure, particularly in cases with coagulopathy
Pregnancy/postpartum status
Bacterial endocarditis
History of severe contrast allergy or hypersensitivity

**Table 3:** Intra-arterial thrombolytic therapy and approved regimens in ALI.

Thrombolytic	Doses and Regimen	Comments
Streptokinase	50.000–120.000 IU over 4 h, followed by 1000–8000 IU/h	UFH 600 IU/h [27]
Urokinase	4000 IU/min or 250.000 IU bolus, followed by 4000 IU/h for 4 h, then 2000 IU/h (max 36 h)	UFH 600 IU/h [25,26]
Alteplase	1–2 mg bolus, followed by 0.05 mg/kg/h	UFH 10.000 IU/24 h [25]
